# Dispersant Molecules with Functional Catechol Groups for Supercapacitor Fabrication

**DOI:** 10.3390/molecules26061709

**Published:** 2021-03-19

**Authors:** Kaelan Rorabeck, Igor Zhitomirsky

**Affiliations:** Department of Materials Science and Engineering, McMaster University, Hamilton, ON L8S4L7, Canada; rorabeck@mcmaster.ca

**Keywords:** catechol, manganese dioxide, carbon nanotube, composite, dispersant, supercapacitor

## Abstract

Cathodes for supercapacitors with enhanced capacitive performance are prepared using MnO_2_ as a charge storage material and carbon nanotubes (CNT) as conductive additives. The enhanced capacitive properties are linked to the beneficial effects of catecholate molecules, such as chlorogenic acid and 3,4,5-trihydroxybenzamide, which are used as co-dispersants for MnO_2_ and CNT. The dispersant interactions with MnO_2_ and CNT are discussed in relation to the chemical structures of the dispersant molecules and their biomimetic adsorption mechanisms. The dispersant adsorption is a key factor for efficient co-dispersion in ethanol, which facilitated enhanced mixing of the nanostructured components and allowed for improved utilization of charge storage properties of the electrode materials with high active mass of 40 mg cm^−2^. Structural peculiarities of the dispersant molecules are discussed, which facilitate dispersion and charging. Capacitive properties are analyzed using cyclic voltammetry, chronopotentiometry and impedance spectroscopy. A capacitance of 6.5 F cm^−2^ is achieved at a low electrical resistance. The advanced capacitive properties of the electrodes are linked to the microstructures of the electrodes prepared in the presence of the dispersants.

## 1. Introduction

Organic molecules, containing catechol groups, exhibit exceptionally strong adsorption on inorganic surfaces, which is a key factor for their applications for surface modification of various materials and fabrication of adherent coatings [1]. The adsorption mechanism of such molecules is similar to that of mussel proteins bonding to different surfaces, which results in super strong adhesion [2,3,4]. It is based on the bidentate chelating or bridging bonding of phenolic OH groups of the catechol ligands [1] to the metal atoms. The structural features of catecholates have rendered them useful in dispersion of various inorganic materials and fabrication of coatings by electrophoretic deposition [1,5]. Various charged dispersants have been developed for nanotechnology of functional materials [1]. The use of catecholate bonding mechanism has been gaining ground in the development of liquid-liquid extraction techniques [6], which facilitate the fabrication of non-agglomerated nanoparticles for diverse applications. Polyaromatic catecholates allowed efficient co-dispersion of inorganic materials and carbon nanotubes for the fabrication of advanced composites [7,8]. Various catecholate molecules were used as capping and structure directing agents for the synthesis of non-agglomerated nanoparticles, coated particles and nanorods with high aspect ratios [9,10,11,12,13,14]. Moreover, it was found that metastable materials can be synthesized in the presence of catecholate molecules [15].

Significant interest has been generated in the synthesis of polymer adhesives, containing catecholate monomers [16,17,18] and modification of polymers with catechol molecules [19,20]. The adsorption of catecholates on semiconductors allowed for enhanced charge transfer, advanced optical and photovoltaic properties, which were used for the development of various photovoltaic devices and sensors [21,22,23]. Catecholate molecules have been utilized for the fabrication of magnetic nanoparticles with enhanced magnetization [24], materials with luminescent properties [25], and quantum dots [26].

Anionic catecholate molecules were utilized for the fabrication of polypyrrole coatings on various non-noble substrates by anodic electropolymerization [27,28,29]. The role of catecholates in the electropolymerization process was multifunctional. Such molecules acted as anionic dopants for the electropolymerization process, facilitated charge transfer and allowed for electropolymerization at reduced electrode potential, which is critically important for corrosion prevention and fabrication of adherent coatings. Catecholate molecules facilitated incorporation of carbon nanotubes into the polypyrrole coatings [29]. Moreover, the catecholate-type bonding of the molecules to the electrode surface was another important factor for the fabrication of adherent polypyrrole coatings. Antifouling polymer coatings were prepared using catecholate molecules as anchors and initiators for surface-initiated polymerization on metallic substrates [30]. The strong adsorption of catecholates on metal surfaces was an important factor for their applications as corrosion inhibitors for stainless steel [31].

Many applications of catecholates are based on their interesting redox properties [32,33,34]. Catecholate molecules were used as reducing agents for the synthesis of inorganic nanoparticles by chemical precipitation methods [20,32]. Moreover, there is a growing interest in the applications of redox active catecholates for the fabrication of electrochemical sensors [19,20] and supercapacitors [35]. Chiral catecholate molecules were utilized for the fabrication of sensors for chiral electrochemical recognition of biomolecules [36]. Recently it was discovered that catecholate molecules can be used as charge transfer mediators between charge storage material and current collector of supercapacitor electrodes [9]. As a result, significant improvement in charge storage properties was achieved [9].

The increasing number of successful applications of catecholates and promising results achieved in various research fields have generated interest in fundamental investigation of various catecholate molecules. This interest is fueled by the rich functional properties of catecholate molecules. The investigation of multifunctional catecholate molecules, combining properties of catechol ligands with properties of other functional groups is a promising strategy for the development of advanced materials and composites as well as their surface modification and functionalization. An important task is to analyze the influence of various factors, such as chemical structure and solvent composition on interactions of catecholates with different materials.

Chlorogenic acid and 3,4,5-trihydroxybenzamide are promising molecules for the surface modification of materials by catecholate-type bonding and development of advanced functional materials. Chlorogenic acid is a natural material found in coffee and tea. Previous investigations focused on the rich variety of biomedical and pharmaceutical applications of this molecule [37]. Moreover, chlorogenic acid exhibits interesting functional properties for applications in sensors and photoluminescent devices [38,39,40]. Investigations revealed strong complexation of metal ions with chlorogenic acid [41]. 3,4,5-Trihydroxybenzamide exhibits interesting redox active and antioxidant properties [42,43].

The goal of this investigation was the application of chlorogenic acid and 3,4,5-trihydroxybenzamide for the fabrication of MnO_2_-carbon nanotube (CNT) electrodes for supercapacitors. The approach was based on the catecholate-type bonding of the molecules to the MnO_2_ surface which facilitated particle dispersion and charging. An important finding was the possibility to co-disperse MnO_2_ and CNT in ethanol, which facilitated their enhanced mixing and allowed for the fabrication of advanced electrodes. Testing results showed good capacitive properties at high active mass, which resulted in high areal capacitance.

## 2. Results and Discussion

Recent studies on the development of supercapacitors stressed the importance of advanced manufacturing technologies [44,45,46,47,48]. The development of nanostructured electrodes has generated a need for advanced dispersants for active materials [49,50]. Therefore, in this investigation chlorogenic acid and 3,4,5-trihydroxybenzamide were tested as co-dispersants for MnO_2_ and CNT.

**Figure 1:**
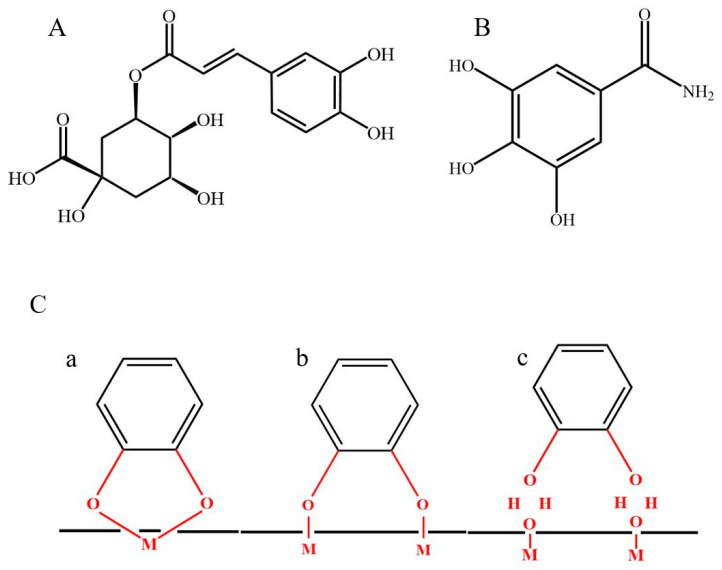
(**A**,**B**) Chemical structures of (**A**) chlorogenic acid and (**B**) 3,4,5-trihydroxybenzamide, (**C**) bonding mechanism of catechol to the metal atoms (M) on inorganic surface: (**a**) chelating, (**b**) inner sphere bridging and (**c**) outer sphere bridging.

Figure 1A,B shows the chemical structures of chlorogenic acid and 3,4,5-trihydroxybenzamide. The structure of chlorogenic acid includes a catechol group. The anionic properties of this molecule are attributed to a carboxylic group. The adsorption of chlorogenic acid on inorganic surfaces can involve catecholate or carboxylate bonding mechanisms [1,51]. However, catecholate bonding to metal oxide surfaces is usually stronger than that of carboxylate bonding [1]. The structure of 3,4,5-trihydroxybenzamide contains a galloyl group, containing three phenolic OH groups bonded to adjacent carbon atoms of the aromatic ring. The galloyl group allows for catecholate type bonding, which usually involves two phenolic OH groups [1]. Moreover, NH_2_ group of the structure can potentially be involved on weak bonding to metal atoms on the inorganic surfaces. Different modes of catecholate bonding are presented in Figure 1(Ca–c), including chelating, bridging inner sphere and bridging outer sphere bonding.

**Figure 2:**
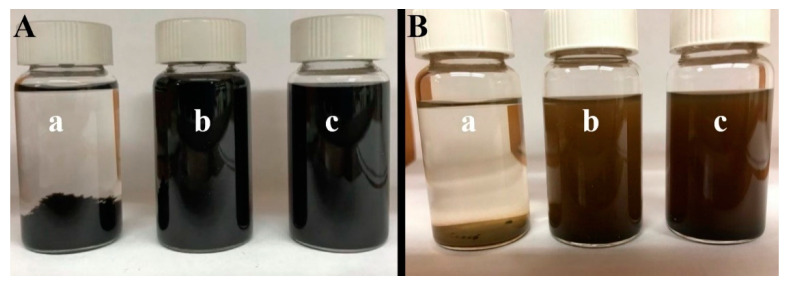
Sedimentation test for (**A**) carbon nanotubes (CNT) and (**B**) MnO_2_ prepared (**a**) without dispersants and in the presence of (**b**) chlorogenic acid and (**c**) 3,4,5-trihydroxybenzamide.

It was suggested that catecholate bonding of chlorogenic acid and 3,4,5-trihydroxybenzamide to the MnO_2_ particle surface will facilitate their dispersion and result in enhanced stability of suspensions for impregnation of current collectors. Sedimentation tests confirmed enhanced stability of the MnO_2_ nanoparticles in ethanol solvent (Figure 2). Figure 2 compares MnO_2_ suspensions prepared without and with dispersant molecules. The enhanced suspension stability achieved in the presence of the dispersants indicates that the dispersants adsorbed on the MnO_2_ particles. Moreover, chlorogenic acid and 3,4,5-trihydroxybenzamide acted as dispersants for CNT in the same solvent. The ability to co-disperse MnO_2_ and CNT in ethanol using chlorogenic acid and 3,4,5-trihydroxybenzamide was critically important for the fabrication of electrodes. Polyvinyl butyral (PVB) was dissolved in the same solvent and obtained slurry, containing MnO_2_, CNT, and PVB binder, was used for the impregnation of Ni foam current collectors. For comparison, the electrodes were prepared using slurries, which were fabricated without dispersants.

**Figure 3:**
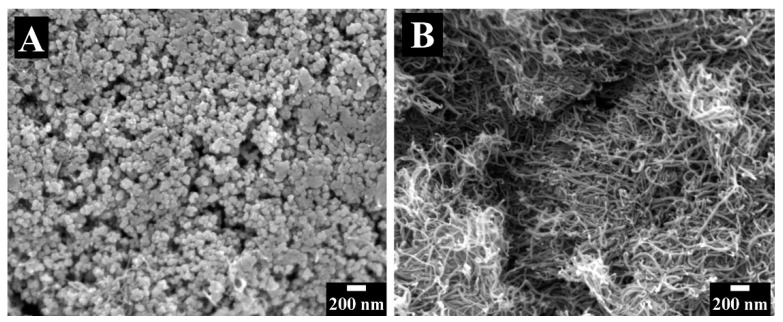
SEM images of electrode prepared without dispersant: (**A**) area of MnO_2_ agglomerate and (**B**) area of CNT agglomerate.

**Figure 4:**
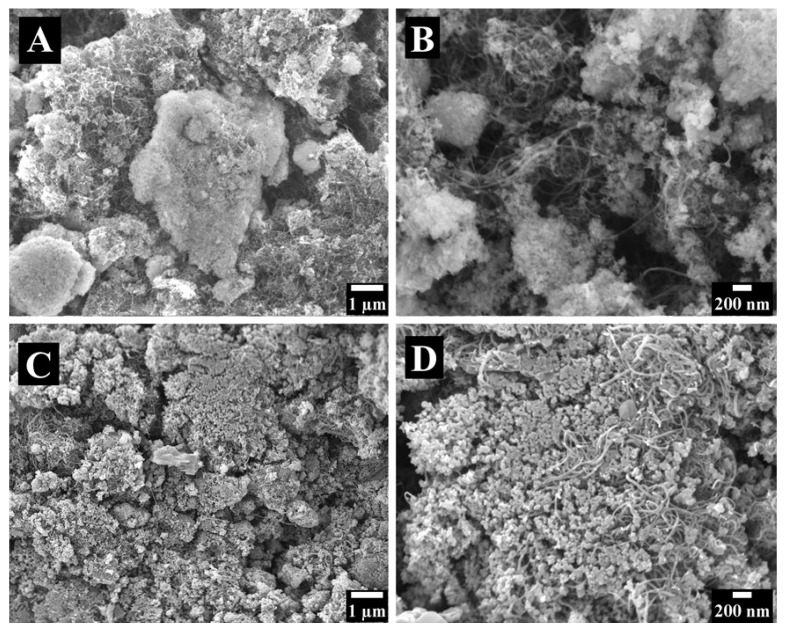
SEM images of different magnifications for electrodes prepared using (**A**,**B**) chlorogenic acid and (**C**,**D**) 3,4,5-trihydroxybenzamide.

Figure 3 shows Scanning electron microscopy (SEM) images of electrodes prepared without dispersants. The electrodes contained large agglomerates of MnO_2_ (Figure 3A) and CNT (Figure 3B). The SEM images indicate poor mixing of the capacitive MnO_2_ material and conductive CNT additives. It will be shown below that such poor mixing resulted in a low capacitance. In contrast, the use of chlorogenic acid and 3,4,5-trihydroxybenzamide dispersants allowed for improved mixing of MnO_2_ and CNT. Figure 4 shows SEM images of MnO_2_-CNT electrodes prepared using the dispersants. The SEM images at low magnifications show porous microstructures, which are beneficial for electrolyte transport. The high magnification images show MnO_2_ particles as well as CNT and indicate enhanced mixing of the components, which allowed for enhanced capacitance.

Capacitance measurements were taken using cyclic voltammetry (CV) and galvanostatic charge–discharge (GCD) methods, which allowed for analysis of integral capacitance in a voltage window of 0 to 0.9 V. Moreover, components of complex differential capacitances were calculated from the electrochemical impedance spectroscopy (EIS) data at a voltage amplitude of 5 mV at different frequencies. The data obtained by different methods provided information on charging behavior of the electrodes at different conditions. The experimental results presented below indicated that capacitance depends on different factors, such has scan rate, frequency, charge/discharge current and voltage or potential.

**Figure 5:**
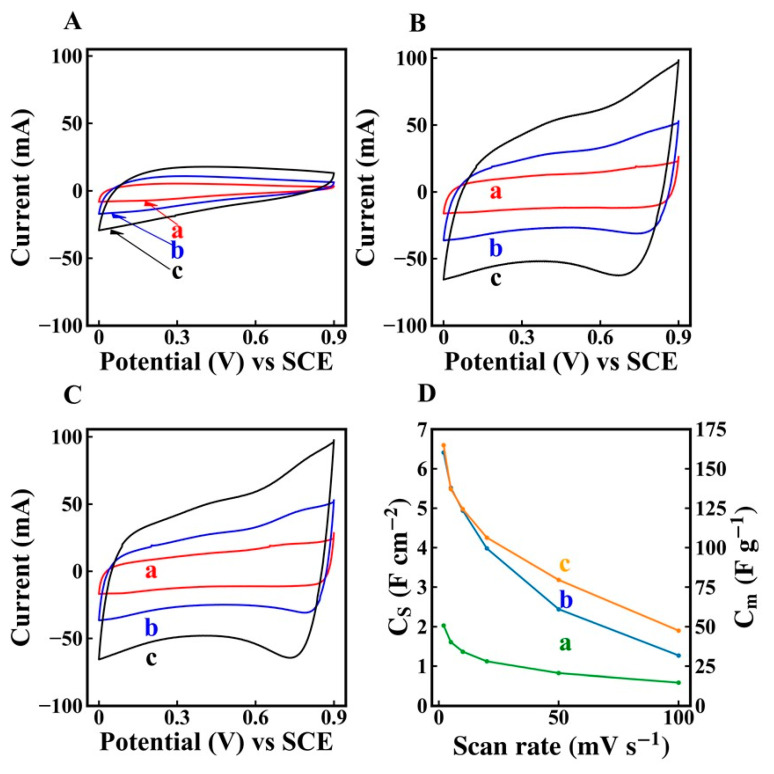
(**A**–**C**) CVs at scan rates of (a) 2, (b) 5 and (c) 10 mV s^−1^ for electrodes prepared (**A**) without dispersant, (**B**) in the presence of chlorogenic and (**C**) in the presence of 3,4,5-trihydroxybenzamide, (**D**) Cs and Cm versus scan rate for electrodes prepared (a) without dispersant, (b) in the presence of chlorogenic acid and (c) in the presence of 3,4,5-trihydroxybenzamide.

**Figure 6:**
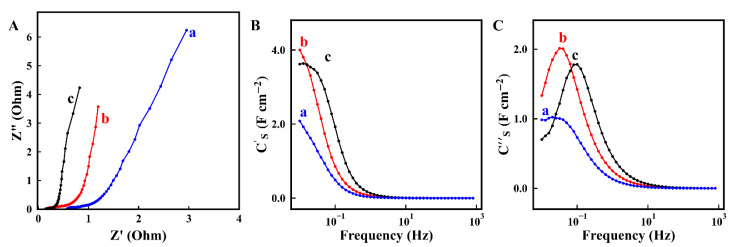
(**A**) Nyquist plot of impedance and (**B**,**C**) components complex capacitance C* = C′ − *i*C″ versus frequency for electrodes prepared (a) without dispersant, (b) in the presence of chlorogenic acid and (c) in the presence of 3,4,5-trihydroxybenzamide.

Figure 5A–C show CV data at different scan rates for electrodes prepared without and with dispersants. The electrodes prepared using dispersants showed significantly larger areas of CVs, which indicated higher capacitances. The integral capacitances in a voltage window of 0 to 0.9 V were measured at different scan rates and presented in Figure 5D. The electrodes prepared with chlorogenic acid, 3,4,5-trihydroxybenzamide and without dispersants showed areal capacitances of 6.4, 6.5 and 2.1 F cm^−2^, respectively at a scan rate of 2 mV s^−1^. The increase in scan rate resulted in the reduced capacitance due to diffusion limitations of an electrolyte in pores. The electrodes prepared using dispersants showed significantly higher capacitance, compared to the electrodes prepared without dispersants. The electrodes, formed using 3,4,5-trihydroxybenzamide, showed higher capacitance retention at 100 mV s^−1^, compared to the electrodes formed using chlorogenic acid. EIS studies (Figure 6) showed higher resistance R = Z′ of the electrodes prepared without dispersant, which resulted from poor mixing of CNT with MnO_2_. Moreover, the electrodes prepared without dispersant showed lower C_S_’ and lower relaxation frequency, corresponding to the C_S_″ maximum. The electrodes prepared in the presence of chlorogenic acid showed the highest capacitance at 10 MHz. However, the electrodes formed using 3,4,5-trihydroxybenzamide showed higher capacitance at frequencies above 50 Hz, indicating better charge storage properties at higher charge discharge rates in agreement with CV data. The electrodes prepared using 3,4,5-trihydroxybenzamide as a dispersant showed the lowest resistance and the highest relaxation frequency, as indicated by the location of the C’’ maximum. It is important to note that capacitances, calculated from the CV data, depended on scan rate, whereas the capacitances calculated from the impedance data depend on frequency. The comparison of the capacitances calculated at the same charge–discharge time scale showed that integral capacitances were higher than differential capacitances. The difference in the differential and integral capacitance was discussed in the literature [52]. It was shown that such difference can result from various reasons, such as the presence of sites with different redox potentials and limited access of the electrolyte to some redox sites at low voltages [52]. Testing results showed beneficial effect of improved mixing of MnO_2_ and CNT, which was achieved using dispersant molecules. However, this investigation revealed some difference in electrochemical behavior of the electrodes prepared using chlorogenic acid and 3,4,5-trihydroxybenzamide. Turning again to the chemical structures of the molecules (Figure 1) it is seen that the dissociation of the carboxylic group of adsorbed chlorogenic acid can potentially impart a negative charge to MnO_2_ and CNT and improve their dispersion and mixing. This can result in better performance of the composite electrodes, which was observed at low frequencies. However, little attention has been paid in the available supercapacitor literature to possible local pH changes at the positive electrode during charge–discharge process.

It is known that the application of a positive potential to the electrode usually results in a local pH decrease [53]; therefore, the protonation of amino groups of organic molecules [1,53] can be expected at such conditions. Therefore, it was hypothesized that the protonation of NH_2_ groups of adsorbed 3,4,5-trihydroxybenzamide can potentially impart a positive charge to the electrode material.

The charging process of the MnO_2_ electrode is given by the reaction, involving MnO_2_ oxidation from 3^+^ to 4^+^ and release of adsorbed Na^+^.

MnO_2_Na ↔ MnO_2_ + e^−^ + Na^+^ (1)

It was suggested that the positive charge of the electrodes prepared using 3,4,5-trihydroxybenzamide will facilitate Na^+^ desorption and transport. In contrast, the protonation of the carboxylic groups of the carboxylic acid molecules will result in their discharge. Therefore, it was not surprizing that the electrodes prepared using 3,4,5-trihydroxybenzamide showed lower resistance and better capacitive properties at high scan rates and high frequencies. The results of this investigation indicate that the modification of the charge of an active material by adsorption of organic molecules can potentially open a promising avenue for the development of electrodes with enhanced performance.

**Figure 7:**
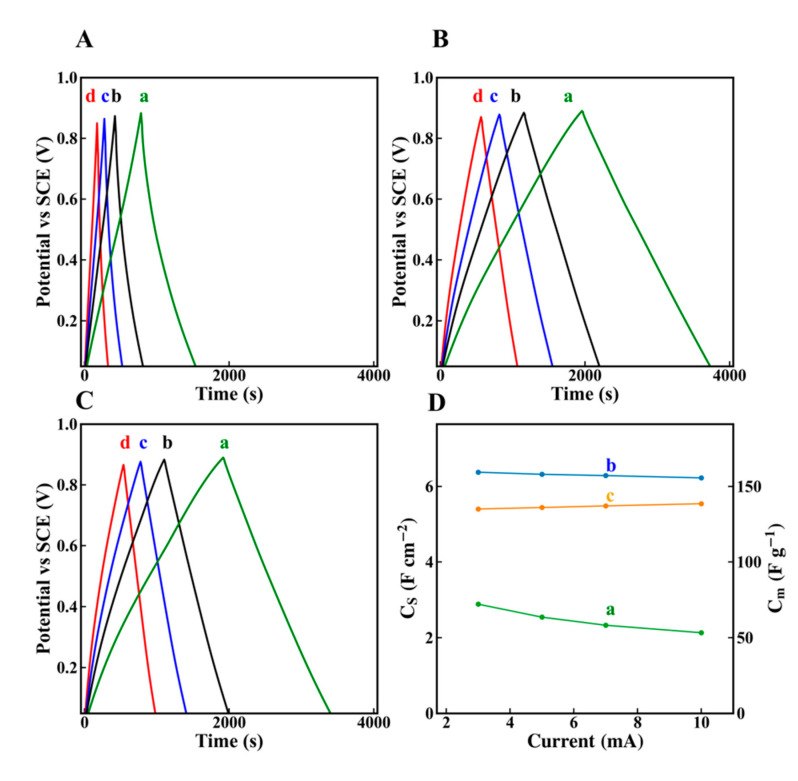
Galvanostatic charge–discharge (GCD) data for electrodes prepared (**A**) without dispersant, (**B**) in the presence of chlorogenic and (**C**) in the presence of 3,4,5-trihydroxybenzamide at current densities of (a) 3, (b) 5, (c) 7 and (d) 10 mA cm^-2^, (**D**) capacitances calculated from the GCD data for electrodes prepared (a) without dispersant, (b) in the presence of chlorogenic and (c) in the presence of 3,4,5-trihydroxybenzamide versus current density.

**Figure 8:**
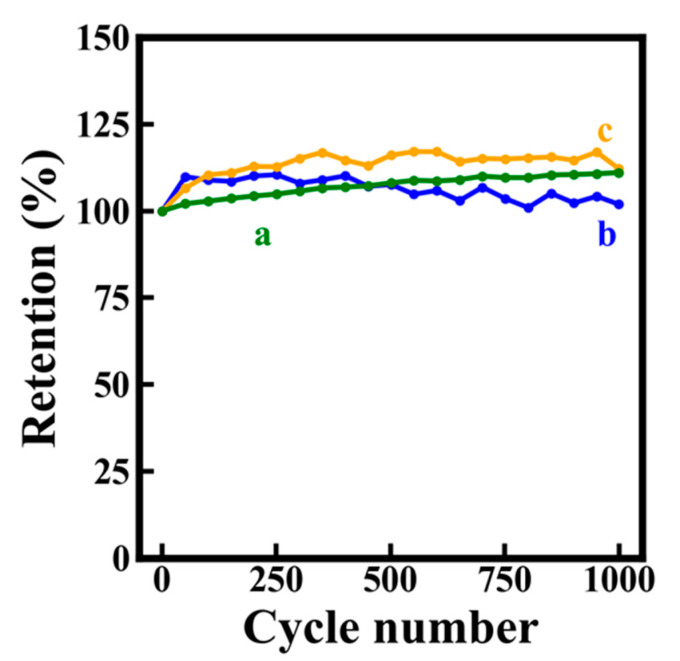
Capacitance retention versus cycle number for electrodes prepared (a) without dispersant, (b) in the presence of chlorogenic and (c) in the presence of 3,4,5-trihydroxybenzamide versus current density.

Figure 7A–C shows GCD data for electrodes prepared without and with dispersants. The use of dispersants resulted in significantly longer charge–discharge currents, which was attributed to higher capacitance. The capacitances calculated from the discharge data were presented in Figure 7D. It is seen that the use of dispersants allowed for the fabrication of electrodes with significantly higher capacitances, compared to the electrodes prepared without dispersants. The electrodes showed good cyclic stability (Figure 8). The electrodes prepared without dispersants, with chlorogenic acid and 3,4,5-trihydroxybenzamide showed capacitance retentions of 111, 102 and 113%, respectively. The slight increase in retention during cycling can result form microstructure changes during cycling or enhanced wetting of the electrodes by the electrolyte [54,55]. Obtained cathodes are promising for applications in asymmetric devices operating in Na_2_SO_4_ electrolyte. However, in order to utilize the benefits of high capacitance of the cathodes in devices, the anodes of similar capacitance must be used. The analysis of literature indicates that reported areal capacitances of some promising anode materials in the same electrolyte are significantly lower and their cyclic stability must be improved [56]. Therefore, further progress must be achieved in the discovery and development of advanced anode materials.

## 3. Materials and Methods

KMnO_4_, polyvinyl butyral (PVB), chlorogenic acid, ethanol, Na_2_SO_4_, and 3,4,5-trihydroxybenzamide (Aldrich), CNT (multiwalled, Bayer) and Ni foams (Vale) were used. MnO_2_ nanoparticles were prepared by a chemical precipitation method described in a previous investigation [49]. This method was based on reduction of Mn^7+^ in an aqueous KMnO_4_ solution by addition of ethanol as a reducing agent. The method resulted in nearly amorphous MnO_2_, which also contained a small amount of a birnessite phase. Previous investigations showed poor stability of prepared MnO_2_ in ethanol. It was found that the dispersant, used in the previous investigation for MnO_2_ dispersion, failed to disperse CNT. Therefore, in this investigation chlorogenic acid, and 3,4,5-trihydroxybenzamide were tested as co-dispersants for MnO_2_ and CNT. The ability to co-disperse MnO_2_ and CNT was critical for the fabrication of slurries, containing dissolved PVB as a binder, for impregnation of Ni foam current collectors and fabrication of high active mass electrodes with the mass of impregnated material of 40 mg cm^−2^. The mass ratio MnO_2_:CNT:PVB was 80:20:3.

SEM studies were performed using a microscope JEOL SEM (JSM-7000F). Cyclic voltammetry (CV) and electrochemical impedance spectroscopy (EIS) studies were performed using a potentiostat-impedance analyzer PARSTAT 2273 (Ametek). EIS data were obtained at an open circuit potential using alternating voltage with an amplitude of 5 mV in the frequency range 0.01–10 kHz. Galvanostatic charge–discharge (GCD) investigations in a fixed potential range were performed using BioLogic VMP 300. Testing was performed using a 3-electrode electrochemical cell containing a working electrode (impregnated Ni foam), counter electrode (Pt mesh), and a reference electrode (SCE, saturated calomel electrode). Aqueous 0.5 M Na_2_SO_4_ solution was used as an electrolyte. Integral capacitances in a potential window of 0 to 0.9 V were calculated from CV and GCD data, as described in previous investigations [56,57]. Mass normalized C_m_ and area normalized C_S_ capacitances were analyzed. Differential capacitance was calculated from the EIS data by the methodology described in [56,57]. All the testing results were obtained for 5 electrodes of the same active mass. The capacitances obtained by the same method for different electrodes varied within 3%.

## 4. Conclusions

For the first time, chlorogenic acid and 3,4,5-trihydroxybenzamide were used as dispersants for MnO_2_ and CNT and fabrication of composite cathodes for supercapacitors. The chemical structures of the molecules facilitated their adsorption on MnO_2_ and CNT, which allowed for co-dispersion and enhanced mixing. Structural peculiarities of the dispersant molecules facilitate dispersion and charging. This simple strategy allowed for the fabrication of supercapacitor electrodes, which showed a capacitance of 6.5 F cm^−2^ and low resistance at high active mass of 40 mg cm^−2^. The analysis of microstructures of electrodes prepared without dispersant and with dispersant provides an insight into the influence of chlorogenic acid and 3,4,5-trihydroxybenzamide dispersants on the electrode performance. The electrodes showed good cyclic stability and can be used for the fabrication of asymmetric supercapacitor devices.

## Data Availability

The data presented in this study are available in: Dispersant molecules with functional catechol groups for supercapacitor fabrication.
